# Electrocardiographic Interference on Bispectral Index Monitor: A Case of Crossed Wires

**DOI:** 10.7759/cureus.29511

**Published:** 2022-09-23

**Authors:** Alexander S Doyal, Hannah E Doyal, David N Flynn, Jay Schoenherr, Jane J Kwon, Priya Kumar

**Affiliations:** 1 Anesthesiology, University of North Carolina at Chapel Hill School of Medicine, Chapel Hill, USA; 2 Medicine, University of North Carolina at Chapel Hill, Chapel Hill, USA; 3 Anesthesiology, University of North Carolina, Chapel Hill, USA; 4 Anesthesiology, University of North Carolina School of Medicine, Chapel Hill, USA

**Keywords:** electrocardiogram (ecg/ekg), electrocardiogram, monitoring interference, neuro-monitoring, bis, bispectral index monitoring

## Abstract

The Bispectral Index (BIS) has been widely utilized to monitor patients’ levels of consciousness during anesthesia. Despite its practicality and prevalence, BIS monitors have been reported to show erroneous readings due to various factors that interfere with the proper reading of the brain’s electrical activity. We present a case where the BIS monitor misinterpreted the patient’s cardiac activity as her neural activity and resulted in a falsely elevated BIS number despite proper placement and lack of underlying patient medical condition, including neurological injury. It is crucial to remain vigilant about monitoring and understanding BIS readings to assess patients’ awareness and effectiveness of anesthesia properly.

## Introduction

The Bispectral Index (BIS, Aspect Medical Systems, Newton, MA, USA), monitors the patient’s neural response to the hypnotic effect of general anesthesia through a proprietary interpretation of the raw electroencephalogram (EEG). Maintenance of general anesthesia is typically titrated to maintain a BIS numerical rating between the range of 40 and 60. Many different conditions may influence the BIS monitor, leading it to reveal an incorrect state of hypnosis. We will present our case report and then describe various interferences described in the literature.

## Case presentation

A 46-year-old woman (consent acquired) with a past medical history only significant for morbid obesity (body mass index 43), underwent a laparoscopic gastrectomy for weight loss. She was premedicated in the preoperative holding area with 2 milligrams (mg) of midazolam and was then transferred to the operating room. She was pre-oxygenated with 100% oxygen. Anesthesia was induced with 100 micrograms (mcg) fentanyl, 100 mg lidocaine, 200 mg propofol, 16 mg cisatracurium, and 20 mcg dexmedetomidine. She was intubated with a 7.0 endotracheal tube by direct laryngoscopy using a mac 3 laryngoscope. General anesthesia was maintained with sevoflurane and cisatracurium. Higher levels of sevoflurane were used to deepen the anesthetic secondary to the patient’s movement (end-tidal concentration ranging from 1.8% to 3.7%). 

**Figure 1:**
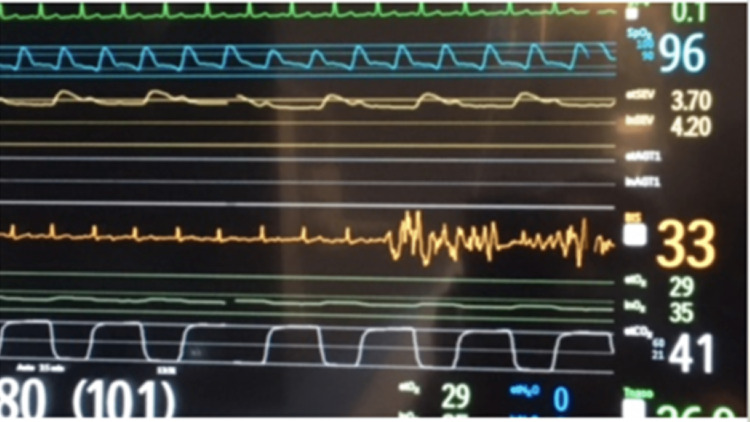
EKG signal being picked up on BIS monitor (orange waveform, numerical value 33)

**Figure 2:**
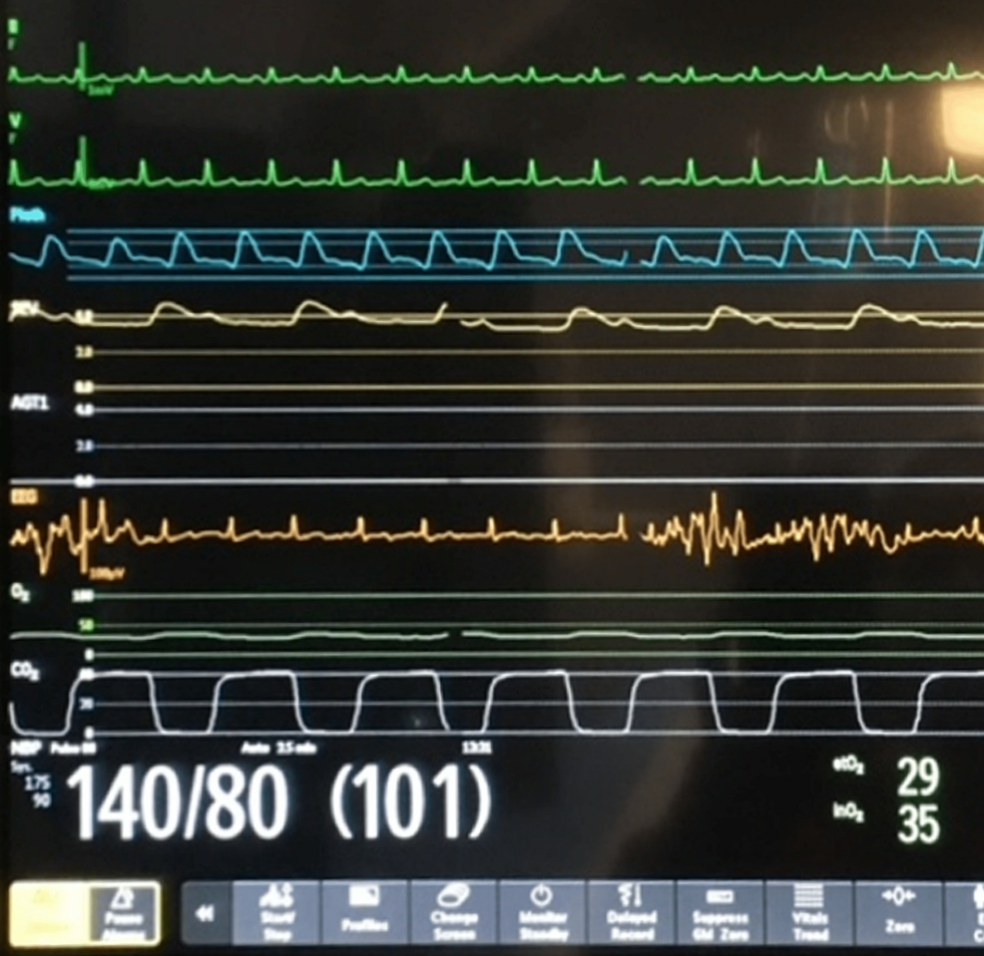
Another example of the EKG signal on the BIS monitor (orange waveform, fifth from top)

The surgery commenced without incidence. A BIS monitor was placed in a typical manner and adequate SQI signals were obtained. At first, the BIS values were in the 40-60 range and were as expected for the patient's depth of anesthesia. However, later on in the surgery, it was noted that the BIS monitor intermittently showed a waveform that mirrored the patient’s electrocardiogram (EKG) activity. This occurred while the end-tidal sevoflurane concentration was particularly high at 3.7%. Under such conditions, the BIS monitor would generally be expected to be low. The clinicians observed that the displayed BIS number appeared to be influenced by the electrical interference of the patient’s EKG (Figures 1, 2).

The surgery proceeded successfully and without complications, BIS values were in the 40-60. At the conclusion of the surgery, the patient was awakened and extubated without incident. The BIS values during this time frame appropriately increased during emergence, progressively climbing into the 80s. She was then transferred to the post-anesthesia care unit (PACU). Her time in the PACU was unremarkable, and she was transferred to the floor. She was discharged home on the following day.

## Discussion

The BIS may be affected by a variety of physiological states of the patient. These include a diverse range of various physiological conditions such as hypoglycemia, hypothermia, cerebral ischemia, the post-ictal state, and low voltage EEG [1]. Pharmacological intraoperative exposure to medications such as ketamine or nitrous oxide will also lead to erroneous BIS readings [1].

The BIS monitor may also be affected by external influences. The BIS constantly evaluates the electrical activity of the brain by interpreting the faint signals of the EEG. Because the monitor is in essence observing small electrical waveforms, it may be susceptible to external electromagnetic interference. The operating room is a rather electrically noisy environment. All electrical equipment produces a field of electromagnetic radiation to varying degrees.

Several external sources that lead to erroneous data have been identified and previously reported. These include electromagnetic monitoring systems, surgical endoscopic shavers, warming blankets positioned on the patient’s head, noise from cardiopulmonary bypass machines, a patient in deep hypothermic cardiac arrest with isoelectric EEG [2], and electromyographic monitoring endotracheal tubes [3]. These sources of interference should be filtered out and ignored as noise by the BIS computational unit. However, this interference may also be interpreted as the patient’s actual EEG signals, leading to an erroneously high BIS number [1].

The appearance of the EKG tracing on the BIS monitor has been previously reported in patients with severe neurological injury resulting in burst suppression and an isoelectric EEG in the intensive care unit [4,5]. In the absence of neurological electrical activity, the BIS erroneously reported cardiac electrical activity as cortical in origin. This could have led to the providers thinking that a brain-dead patient continued to have some neurological function. In another example, a display of cardiac activity by a BIS monitor was observed when the BIS is not placed on the forehead, as seen by Suzuki et al. They used an atypical periauricular placement of the BIS monitor low on the patient’s face. Of note, this is not how the monitor is designed to be used. This method of positioning places the electrodes much further from the electrical impulses of the brain and nearer to the heart [6]. It is not surprising that in this location the cortical signal was weaker and the cardiac electrical signals much stronger. Electrical activity generated by an implanted cardiac pacemaker has also been shown to interfere with BIS monitors, resulting in erroneously high readings [7,8]. This may be due to the high electrical signal of the pacemaker compared to the intrinsic electrical activity of the heart. We report the appearance of cardiac electrical activity on a correctly placed BIS monitor in a neurologically intact patient under general anesthesia.

## Conclusions

BIS monitoring is an important tool used by anesthesiologists to help titrate anesthetics to an appropriate depth. Even though this modality is generally reliable, anesthesia providers must be aware of potential sources of interference that can result in erroneous readings. The findings of this case report further highlight the notion that when monitoring the BIS as an indicator of the depth of anesthesia, much caution is needed. Many external sources of electrical “noise” may be interpreted as cortical “signals” and confuse the monitor. Misinterpreting these signals may lead to underestimating the depth of anesthesia. The subsequent overdosing of anesthetics may lead to patient harm. In this specific case, the patient was deeply anesthetized. This may have contributed to low EEG amplitudes that allowed for the relatively low energy signals of the patient’s cardiac cycle to be picked up and interpreted as true EEG signals. During surgery, close attention to the EEG waveform is necessary, as simply reading the number may lead to erroneous conclusions about the patient’s actual state of consciousness.
